# Immunomodulatory parasites and toll-like receptor-mediated tumour necrosis factor alpha responsiveness in wild mammals

**DOI:** 10.1186/1741-7007-7-16

**Published:** 2009-04-22

**Authors:** Joseph A Jackson, Ida M Friberg, Luke Bolch, Ann Lowe, Catriona Ralli, Philip D Harris, Jerzy M Behnke, Janette E Bradley

**Affiliations:** 1 School of Biology, University of Nottingham, University Park, Nottingham, NG7 2RD, UK; 2 School of Biological Sciences, University of Liverpool, Biosciences Building, Crown Street, Liverpool, L69 7ZB, UK

## Abstract

**Background:**

Immunological analyses of wild populations can increase our understanding of how vertebrate immune systems respond to 'natural' levels of exposure to diverse infections. A major recent advance in immunology has been the recognition of the central role of phylogenetically conserved toll-like receptors in triggering innate immunity and the subsequent recruitment of adaptive response programmes. We studied the cross-sectional associations between individual levels of systemic toll-like receptor-mediated tumour necrosis factor alpha responsiveness and macro- and microparasite infections in a natural wood mouse (*Apodemus sylvaticus*) population.

**Results:**

Amongst a diverse group of macroparasites, only levels of the nematode *Heligmosomoides polygyrus *and the louse *Polyplax serrata *were correlated (negatively) with innate immune responsiveness (measured by splenocyte tumour necrosis factor alpha responses to a panel of toll-like receptor agonists). *Polyplax serrata *infection explained a strikingly high proportion of the total variation in innate responses. Contrastingly, faecal oocyst count in microparasitic *Eimeria *spp. was positively associated with innate immune responsiveness, most significantly for the endosomal receptors TLR7 and TLR9.

**Conclusion:**

Analogy with relevant laboratory models suggests the underlying causality for the observed patterns may be parasite-driven immunomodulatory effects on the host. A subset of immunomodulatory parasite species could thus have a key role in structuring other infections in natural vertebrate populations by affecting the 'upstream' innate mediators, like toll-like receptors, that are important in initiating immunity. Furthermore, the magnitude of the present result suggests that populations free from immunosuppressive parasites may exist at 'unnaturally' elevated levels of innate immune activation, perhaps leading to an increased risk of immunopathology.

## Background

Natural vertebrate populations are chronically exposed to multiple infections and experience a range of environmental stressors. The vertebrate immune system will have evolved in ancestral populations subject to these pressures. Given that our understanding of mammalian immunology is largely based on rodents reared under highly unnatural pathogen- and stress-free conditions, analysing immune responses in wild populations may give crucial insights into how the immune system functions in its natural context. There may be fundamental implications for how we understand immunological dysfunction observed in modern humans. Across recent generations, humans in developed countries, much like laboratory mouse strains, have been exposed to a very different profile of infections to that encountered by their ancestors. At the same time there is evidence of a continuing breakdown of immune regulation in humans in industrialised societies, manifested by increases in allergic [1] and autoimmune disorders [2]. A putative functional link between reduced infection exposure and immune hyperactivity in human populations (the 'hygiene hypothesis') has long been proposed [3]. A more recent refinement of this idea is that metazoan infections may lead to the healthy development of immunoregulatory networks due to their ability to stimulate expansions of regulatory T-cells [4]. The evolutionary basis for this is that strong immunomodulatory stimuli from metazoan parasites have been a constant presence during vertebrate history, and that the immune system is now maladapted to a situation where these organisms are absent [5]. This is consistent with the considerable evidence that helminth infections of humans stimulate immunosuppressive effects and increased T-regulatory activity [6-9]. However, support for the evolutionary role of parasite-induced immunoregulation would be strengthened if immune suppression by metazoan parasites could be shown to also be a general phenomenon in wild vertebrate populations. Studies in wildlife systems might also give greater insight into the range of parasitic organisms involved and the ecological context in which immunosuppression occurs.

The analysis of immune responses in wildlife has been hindered by a lack of specific reagents for the measurement of immunological molecules in non-model organisms [10]. Here we take advantage of conservation in the murine pro-inflammatory signalling molecule tumour necrosis factor alpha (TNF-α) to develop a novel assay of innate toll-like receptor (TLR)-mediated immune responsiveness in a wild rodent population. TLRs are pattern-recognition receptors (PRRs) that detect conserved molecular patterns associated with certain types of pathogenic organism or with endogenous 'danger' signals [11]. Currently, TLRs are thought to be the most important family of PRRs in initiating innate immunity and the activation of antigen presenting cells that trigger adaptive responses. Their central role in the immune system gives a number of potential advantages in assays which seek to provide an overall measure of immune function. As there is a diversity of TLRs [12] with differing ligand specificities, responses for a panel of different receptor-ligand combinations may give indications of immune responsiveness towards a broad range of infections. In addition to triggering and influencing the phenotypic trajectory of innate and adaptive immunity, TLR responses are also themselves influenced by signals from many other elements of the immune system, including signals from regulatory adaptive responses [13]. The assay that we describe below may therefore offer a relatively holistic index of overall immune activation.

We used this assay of innate immune responses to analyse a cross-sectional natural population sample of the wood mouse, *Apodemus sylvaticus*, in the UK and test hypotheses of covariation between innate immune activation, parasitic infection and other host variables. On the basis of our results we suggest that a subset of metazoan parasites can have strong suppressive effects on innate immunity that over-ride effects due to individual body condition or reproductive status.

## Results

### TLR-response profile

**Figure 1:**
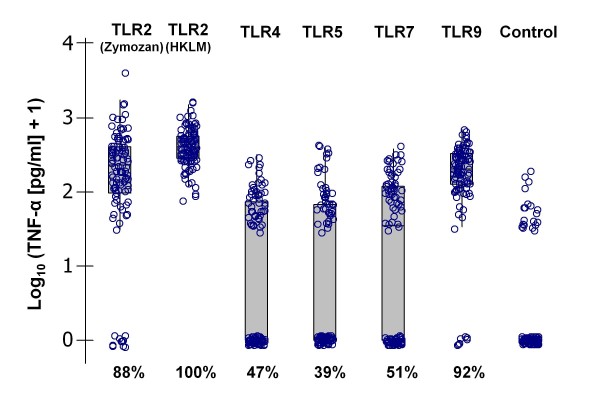
**Scatter of individual *ex vivo *splenocyte tumour necrosis factor alpha responses to a panel of toll-like receptor agonists (Table 1)**. Box plots show the individual scatter of log-transformed data, the median (line), the interquartile range (box) and the range between the highest and lowest values (whiskers) excluding outliers (values differing by more than 1.5 times the interquartile range interval from the nearest limit of the interquartile range). Percentages given below each plot relate to the number of individuals producing an above-control response.

We measured *ex vivo *TNF-α accumulations in splenocyte cultures exposed to a panel of defined ligands for TLRs 2, 4, 5, 7 and 9 (Table 1). For each animal these stimulatory assays provided a profile of pro-inflammatory responses mediated by individual TLRs that might reflect the underlying phenotype of innate immune activation. Great inter-individual variability in responsiveness was detected (Figure 1). Across the sample of 100 *A. sylvaticus*, the strongest mean responses were against ligands for TLR2 and TLR9, for which most (>90%) individuals showed above-control responses. Above-control responses were seen against TLR 4, 5 and 7 ligands (Figure 1) in, respectively, 47, 39 and 51% of all individuals. For all TLR ligands examined, TNF-α production was significantly elevated in treated compared with control splenocytes at the level of the entire sample of *A. sylvaticus *(Sign tests, *P *< 0.0005).

**Table 1:** Details of toll-like receptors (TLRs) stimulated in cellular assays.

**Receptor**	**Site of expression**	**Agonists**
TLR2	Cell surface	Fungal zymosan (10 μg/ml)
		Heat-killed *Listeria monocytogenes *(0.6 × 10^8 ^cells/ml)
TLR4	Cell surface	*Escherichia coli *K12 lipopolysaccharide (3 μg/ml)
TLR5	Cell surface	*Salmonella typhimurium *flagellin (0.6 μg/ml)
TLR7	Endosomal	Imiquimod (1.5 μg/ml)
TLR9	Endosomal	Oligonucleotide ODN2006 (6 μg/ml)

### Positive covariation amongst TLR-mediated responses

**Figure 2:**
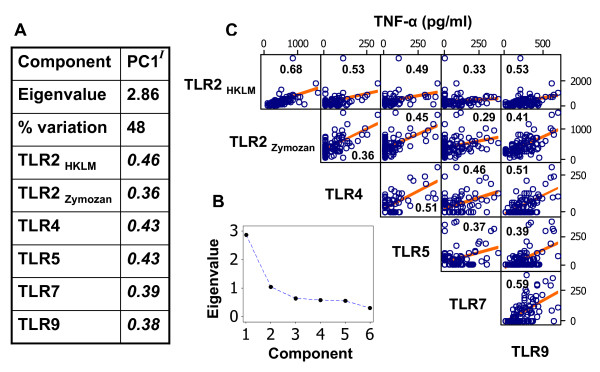
**Strong positive covariation amongst toll-like receptor (TLR)-mediated responses**. A) A principal components analysis (PCA) of log-transformed TLR-mediated responses showed a dominant first component (PC1^I^) accounting for 48% of total variation. For each TLR-mediated response, PC1^I ^coefficients (shown alongside) are of similar magnitude and the same sign, indicating a general pattern of positive association. B) Scree plot of PCA eigenvalues, showing the predominance of the first component. C) Matrix of bivariate scatterplots between untransformed TLR-mediated responses with non-parametric correlation coefficient (Spearman's) and least squares regression line shown for reference.

Amongst individual *A. sylvaticus *there was strong positive intercorrelation between responses mediated through different TLRs (Figure 2). The first component (*P *< 0.001) extracted from a principal components analysis (PCA) of the six TLR-response variables showed large coefficients of the same sign and accounted for 48% of total variation. Pairwise Spearman's correlation coefficients (*r*_*s*_) for individual variables were also all positive and highly significant (*P *< 0.0005, or *P *= 0.001 in the case of TLR2 versus TLR7 responses), with *r*_*s *_of between 0.29 and 0.68. These significant positive relationships were maintained when pairwise relationships between TLR-response variables were examined in linear mixed models in which the fixed model component was used to account for confounder variables and the random model component was used to account for data non-independence introduced by the immunological assaying procedures (see Methods) (not shown).

### Parasite community structure

**Figure 3:**
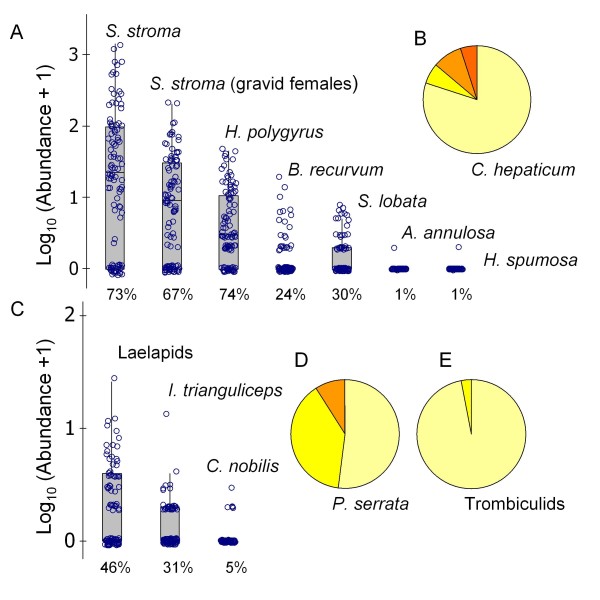
**Amongst-host distributions of metazoan parasites in *Apodemus sylvaticus *(*N *= 100) at Cotgrave Forest, UK (+52.891, -1.041242) July–November 2007**. A) Box plots of log-transformed individual counts for endoparasitic helminths occurring in the gut (*Syphacia stroma*, *Heligmosomoides polygyrus*, *Brachylaemus recurvum*, *Skrjabinotaenia lobata*, *Aoncotheca annulosa*, *Heterakis spumosa*). For *S. stroma*, results are presented for all stages and for gravid females alone. Percentage prevalence is given below each box plot. B) Pie chart representing proportional distribution of the *A. sylvaticus *sample amongst infection grades for the hepatozoic nematode *Calodium hepaticum *(see Methods). C) Box plots of log-transformed individual counts for laelapid mites, ticks (*Ixodes trianguliceps*) and fleas (*Ctenophthalmus nobilis*) occurring on the body surface. Percentage prevalence is given below each box plot. D-E) Pie charts representing proportional distribution of the *A. sylvaticus *sample amongst infection grades for lice (*Polyplax serrata*) (D) and trombiculid mites (E) (see Methods). Box plots show the individual scatter of data, the median (line), the interquartile range (box) and the range between the highest and lowest values (whiskers) excluding outliers (values differing by more than 1.5 times the interquartile range interval from the nearest limit of the interquartile range). The pie charts show increasing infection grade clockwise.

**Figure 4:**
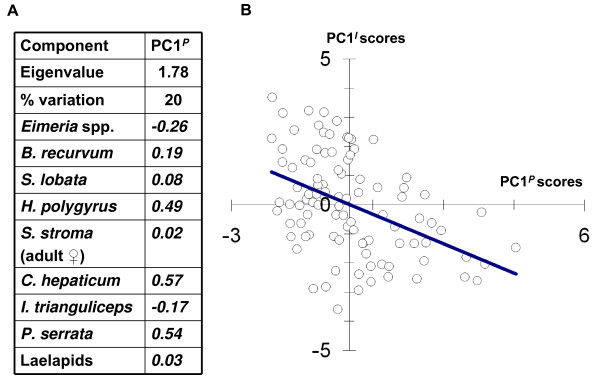
**Structure in the parasite community and its relationship to toll-like receptor (TLR)-mediated responses**. A) Limited structuring of the parasite community. Principal components analysis (PCA) of infection variables showed a first component (PC1^*P*^) accounting for 20% of total variation. Large PC1^*P *^positive coefficients (shown alongside individual infection variables) occurred for *Heligmosomoides polygyrus*, *Calodium hepaticum *and *Polyplax serrata*, indicating a pattern of positive covariation between these species. At the same time, a moderate negative coefficient for *Eimeria *spp. faecal oocyst count indicated a possible contrasting trend in coccidial oocyst shedding. Coefficients for *Brachylaemus recurvum*, *Skjrabinotaenia lobata*, *Syphacia stroma*, *Ixodes trianguliceps *and laelapid mites were small, indicating a lack of influence on PC1^*P*^. B) Strong association between infection status and toll-like receptor (TLR)-mediated responsiveness. When PC1^*P *^scores were used to represent a major aspect of variation in the parasite community and PC1^*I *^scores from a PCA of log-transformed TLR-mediated responses (see Figure 2) were used to represent overall innate immune responsiveness, there was a strong confounder-adjusted relationship between the two variables (general linear mixed model, *P *< 0.001). Plot shows the scatter of individual observations with a least square regression line for reference.

Quantitative infection variables are summarised in Figure 3. Correlational structure amongst these was relatively weak and mainly linked to host size. The first component from a PCA analysis (PC1^*p*^) of the common species, although highly significant (*P *= 0.001), only explained 20% of total variation. PC1^*p *^was dominated by large coefficients of the same sign for *Heligmosomoides polygyrus*, *Calodium hepaticum *and *Polyplax serrata *(Figure 4A), indicating a pattern of positive covariation between these species. To a lesser extent, a moderate negative coefficient for *Eimeria *faecal oocyst count (FOC) indicated a tendency for *Eimeria *spp. fecundity to covary negatively. However, when pairwise species associations were assessed by non-parametric correlation coefficients, there were only significant positive relationships between *H. polygyrus *and *C. hepaticum *(*P *= 0.001), *H. polygyrus *and *P. serrata *(*P *= 0.037) and *C. hepaticum *and *P. serrata *(*P *< 0.0005). Further analysis by generalised linear models (GLMs) suggested that no significant inter-species associations remained when spatio-temporal and host variables were accounted for, except for a marginal positive relationship of *P. serrata *to *C. hepaticum *infection (*P *= 0.041). Most correlative structure amongst the infection variables appeared to be related to SVL (snout-vent length), which might reflect changes in susceptibility and/or exposure with host age. There were positive trends with SVL for *Skrjabinotaenia lobata *(GLM, *P *= 0.004), *C. hepaticum *(*P *= 0.010), *H. polygyrus *(*P *= 0.004) and *P. serrata *(*P *= 0.049) and a negative trend for laelapids (*P *= 0.039). Some species showed seasonal variation: *Ixodes trianguliceps *increased during the autumn (*P *= 0.002) and *Brachylaemus recurvum *decreased (*P *= 0.011). Other features in the data were tendencies for *H. polygyrus *to occur more often within some spatial trap groups (*P *= 0.021) and (as previously reported [14]) for *Syphacia stroma *to occur more frequently in males (*P *= 0.002).

### Associations between infection variables and TLR-mediated responses

**Figure 5:**
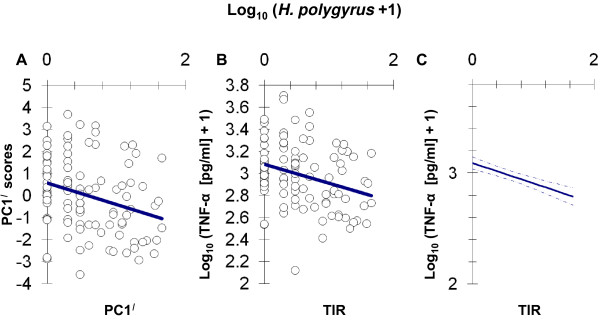
**Negative relationship of toll-like receptor (TLR)-mediated responses with *Heligmosomoides polygyrus *abundance**. A) Scatter of first principal component (PC1^*I*^) scores from a principal components analysis (PCA) of log-transformed TLR-mediated responses against log-transformed *H. polygyrus *abundance. Least squares regression line shown for reference. B) Scatter of log-transformed total TLR-mediated tumour necrosis factor alpha (TNF-α) response (summed across all six TLR variables), TIR, against log-transformed *H. polygyrus *abundance. Least squares regression line shown for reference. C) General linear mixed model prediction of the relationship (solid line) between log-transformed *H. polygyrus *abundance and log-transformed total TNF-α production. Dashed lines indicate 1 standard error above and below.

**Figure 6:**
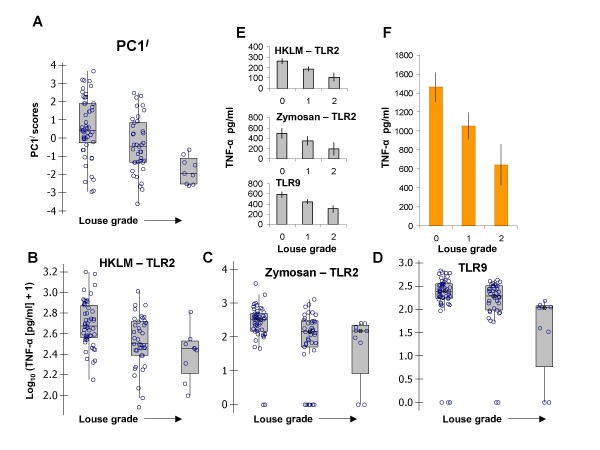
**Negative relationship of toll-like receptor (TLR)-mediated responses with louse (*Polyplax serrata*) infection grade**. A-D) Scatter of TLR-response variables at increasing louse infection grades. Box plots show the individual scatter of data, the median (line), the interquartile range (box) and the range between the highest and lowest values (whiskers) excluding outliers (values differing by more than 1.5 times the interquartile range interval from the nearest limit of the interquartile range). A) Overall TLR-mediated responsiveness represented by first principal component (PC1^*I*^) scores from a principal components analysis (PCA) of the six log-transformed TLR variables. B) Log-transformed TLR2 response against HKLM. C) Log-transformed TLR2 response against zymosan. D) Log-transformed TLR9 response against ODN2006. E-F) Confounder-adjusted general linear mixed model predictions for the effect of louse grade on untransformed TLR-mediated tumour necrosis factor alpha (TNF-α) responses (pg/ml) with other model terms averaged. One standard error is shown above and below each estimate. E) TLR2 and TLR9-mediated responses. F) Total TLR-mediated response (summed across all six TLR variables), TIR.

To test our main hypothesis, that innate immune responsiveness is related to infection variables, we initially used summary variables (PC1 scores) derived from the PCAs described above to represent the strongest patterns of multivariate variation in the immunological and parasitological datasets. Given the tendency for different TLR responses to co-vary positively, first component scores (PC1^*I*^) from the PCA of TLR responses were used to represent overall innate immune responsiveness. A limited simplification of the parasite data was also possible by using first principal component scores (PC1^*p*^) to reflect positive covariation between *P. serrata*, *H. polygyrus *and *C. hepaticum *infection and a tentative contrasting trend in *Eimeria *spp. oocyst shedding. General linear mixed model (LMM) analysis, accounting for confounder variables and correlations introduced by the immunological assaying procedures, indicated that PC1^*I *^was very highly significantly negatively related to PC1^*p *^(*F*_1,83.4 _= 16.52; *P *< 0.001) (Figure 4B). *Post-hoc *analysis (LMMs) of all individual infection variables for common parasite species (prevalence ≥ 20%) suggested a significant negative relationship of PC1^*I *^to log-transformed *H. polygyrus *individual counts (*F*_1,83.7 _= 7.80; *P *= 0.006) and *P. serrata *infection grade (0, absent; 1 <20 egg cases present; 2, >20 egg cases present) (*F*_1,84.4 _= 11.17; *P *= 0.001)(Figures 5 and 6). PC1^*I *^was significantly positively related to *Eimeria *spp. prevalence (*F*_1,78.6 _= 4.48; *P *= 0.037) but was not associated with *B. recurvum*, *S. lobata*, *S. stroma*, *C. hepaticum*, laelapids and *I. trianguliceps*. Analysis of individual infection variables with respect to individual TLR-response variables in LMMs or generalised linear mixed models (GLMMs) suggested that there was a highly significant negative relationship of *H. polygyrus *abundance to TLR2/zymosan response (LMM, *F*_1,83.1 _= 11.78, *P *< 0.001) and weaker negative relationships to TLR2/heat-killed *Listeria monocytogenes *(HKLM) response (LMM, *F*_1,76 _= 4.65; *P *= 0.034), TLR5 response (GLMM, *F*_1,86.6 _= 3.10; *P *= 0.082) and TLR9 response (LMM, *F*_1,77.3 _= 5.14; *P *= 0.026). Analysis of total TNF-α response summed across all receptor-ligand combinations (TIR) also suggested a significant negative association of *H. polygyrus *abundance and overall TLR responses (LMM, *F*_1,81.1 _= 8.89; *P *= 0.004) (Figure 5B–C). For *Eimeria *spp. prevalence there were significant positive associations with TLR7 response (GLMM, *F*_1,79.2 _= 6.86; *P *= 0.011), TLR9 response (LMM, *F*_1,83.8 _= 4.95; *P *= 0.029) and TIR (LMM, *F*_1,78.1 _= 6.06; *P *= 0.016) that were maintained if log-transformed *Eimeria *FOC was analysed instead of prevalence. Louse infection grade showed highly significant negative association with TLR2 responses to HKLM (LMM, *F*_1,81.1 _= 18.90; *P *< 0.001) and zymosan (LMM, *F*_1,75.0 _= 9.54; *P *= 0.003), TLR9 response (LMM, *F*_1,80.6 _= 7.20; *P *= 0.009) (Figure 6B–D) and TIR (LMM, *F*_1,80.7 _= 17.03; *P *< 0.001). Pairwise analyses of the relationships of the remaining individual parasite variables (common species) to individual TLR-response variables found no significant relationships.

Effect sizes of louse infection grade on the most intense responses (TLR2 and TLR9) and on total TNF-α (summed over all receptor-ligand combinations), TIR, were striking (Figure 6E–F). In LMMs with untransformed TLR variables as the response, the predicted reductions in TNF-α production in mice in the highest louse infection category in relation to mice uninfected with lice were 49% for TLR2/HKLM, 55% for TLR2/zymosan, 59% for TLR9 and 56% for TIR (Table 2).

**Table 2:** General linear mixed model prediction of tumour necrosis factor alpha (pg/ml) responses in louse-free wood mice and wood mice in the heaviest louse infection category.

**Response**	**Uninfected**	**Heavy infection**	**%**Δ^‡^
TLR2/heat-killed *Listeria monocytogenes*	560 (42)	284 (65)	-49.3%
TLR2/zymosan^†^	418 (89)	187 (123)	-55.2%
TLR9	277 (22)	113 (35)	-59.2%
TIR	1463 (144)	647 (214)	-55.8%

Results for model: untransformed tumour necrosis factor alpha (TNF-α) (pg/ml) ~sex + snout-vent length + season + trap group; random model, trap day/process day/assay plate.

^†^The TLR2/zymosan general linear mixed model for untransformed TNF-α was a relatively poor fit to the data, but results for this model, which are highly consistent with model predictions for the other variables, are presented to maintain direct comparability. TIR = total TNF-α (pg/ml) response summed over all receptor-ligand combinations. Standard errors are given in parentheses.

^‡^Percentage change between the uninfected and heaviest infection categories.

### Associations between host variables and TLR-mediated responses

In LMMs excluding parasite variables there was no significant relation of PC1^*I *^to sex (*F*_1,82.2 _= 0.74; *P *= 0.393), SVL (*F*_1,84.8 _= 0.93; *P *= 0.337), or post-capture body weight (BW) (*F*_1,88.1 _= 1.17; *P *= 0.283). A factor reflecting distinct life stages (LH) that would be expected to show differing hormonal backgrounds (juvenile male, non-reproductive adult male, reproductive adult male, imperforate juvenile female, non-pregnant non-lactating adult female, pregnant female) was also unrelated to PC1^*I *^(*F*_4,79.8 _= 0.43; *P *= 0.788). There was no relationship of PC1^*I *^to residuals of BW (BW^*resid*^) (*F*_1,82.6 _= 0.09; *P *= 0.765) and liver weight (LW^*resid*^) (*F*_1,83.8 _= 1.50; *P *= 0.224) from regressions on SVL. No other significant associations were found between individual TLR variables and SVL, BW, BW^*resid*^, LW^*resid *^or LH. Whilst there was a marginally non-significant positive link between packed blood cell volume (PCV) and PC1^*I *^(*F*_1,76.7 _= 3.77, *P *= 0.056) this was principally due to a strong relation of PCV to TLR9-mediated responses (*F*_1,80.9 _= 11.41; *P *= 0.001) but not responses mediated through other receptors (all *P *> 0.05).

## Discussion

Our analysis provides compelling evidence for strong negative associations between some members of the metazoan parasite community and the level of innate immune activation. These were detectable for the nematode *H. polygyrus *and, very strongly, for the louse *P. serrata *but not for other abundant community members. It has long been known from studies of the *Heligmosomoides bakeri*-*Mus musculus *laboratory model that *H. bakeri *can exert non-specific immunosuppressive effects [15,16] that may enable other parasites to survive better in the face of host immunity [17]. More recent studies show it to stimulate expansion of regulatory T-cell subsets [18-21]. Given that TLR-responsiveness can be influenced by regulatory signals from the adaptive immune system [13,22,23] and that some helminth products, including *H. polygyrus *products, also direct the maturation of dendritic cells (DCs) with muted pro-inflammatory properties and down-regulated TLR function [24-28], our current data are consistent with an *H. polygyrus*-dependent depression of innate immunity. Perhaps more surprising is the observation that louse infection is even more strongly associated with innate immune activation. The quantitative effect of this infection was large, with modelling results indicating that the highest levels of louse reproductive activity (>20 louse egg cases per host) were associated with approximately 50% reductions in TLR2 and TLR9-mediated TNF-α responses. This suggests that some ectoparasites that are in contact with the host immune system merely through feeding and attachment at the body surface may exert a stronger influence on the immunoregulatory environment in their host than endohelminth infections. Although *P. serrata *has not been a major focus of recent immunological research, older studies have reported it not only to generate protective immunity in *M. musculus *[29], but also to mediate *in vivo *suppressive effects on T-cell proliferation in some inbred strains of this host [30]. Immunosuppressive effects are well documented in other ectoparasitic arthropods [31,32]. For example, the Salp15 protein secreted in *Ixodes scapularis *saliva can interfere with DC function to reduce TLR-mediated pro-inflammatory cytokine release [33] and directly inhibit CD4^+ ^T-cell activation [34]. It might be predicted that *P. serrata *may also secrete substances into the host that interfere directly with innate immune function or, perhaps by analogy with gastrointestinal nematodes, stimulate expansions of regulatory T-cells. However, a further possibility is that unidentified microbial pathogens (perhaps members of the bacterial genus *Bartonella*?), for which lice might act as a vector, could be responsible for immunosuppressive effects.

Apart from *Heligmosomoides *and lice, no other common metazoan parasites appeared to be associated with variation in innate responsiveness. This may indicate that these species lack immunomodulatory properties in *A. sylvaticus *or occurred at levels too low for the detection of such effects. The cross-sectional study design may also have been unable to detect time-lagged correlations, depending on the relative dynamics of the parasite infrapopulations and immune processes. In the case of *B. recurvum*, *S. lobata *and *C. hepaticum *no experimental data on infection duration and immunology are available to assess this. *Ixodes *ticks, which are known to have immunomodulatory effects in laboratory models (*loc. cit*.), occurred at relatively low levels and the inherently transient nature of host-parasite contacts in these organisms could have compromised the detection of immunological influences. *Syphacia *spp. live and feed in the gut lumen at all stages in the life-cycle and perhaps have less contact with the immune system than nematodes that feed on tissues or have a histozoic phase in their life history. Despite this less intimate association, *Syphacia obvelata *is known to stimulate vigorous Th2 immune responses and down-regulate allergic responses [35] in *M. musculus*. As generation time (1 to 2 weeks) and adult lifespan are very short in *Syphacia *spp. [36,37], instantaneous population size might not reflect the recent history of infection, compromising the ability to detect covariation between worm counts and immune effects.

In contrast to the metazoan parasites, there was a positive association between TLR-mediated TNF-α responses and the presence of microparasitic coccidian infections. This is consistent with a Th1 response and with the immunostimulatory effects reported for *Eimeria *spp. in rodent and avian laboratory models, in which primary infection is typically associated with the production of pro-inflammatory cytokines such as IL-6, TNF-α, IL-12 and IFN-γ [38-40]. Furthermore, a defined *Eimeria *antigen is known to up-regulate pro-inflammatory cytokine production in mice by a MyD88-dependent mechanism, consistent with the involvement of TLRs [41,42].

The parallels between our present immuno-epidemiological results and the parasite-mediated immunosuppressive effects reported in relevant laboratory model studies are suggestive that the patterns we report above are due to parasite causal effects on host immunity. We considered the alternative possibility that stressed individuals in poor condition [43], or individuals in active reproductive states, might make a weaker immune response and therefore have more parasites. Under some circumstances nutritional status can affect immunity and resistance to parasites [44-47] (but see [48]). Glucocorticoids, produced in individuals experiencing high environmental stress, and sex hormones are sometimes [49,50] associated with effects on immunity [44,46]. However, we generally found no associations between the host status variables that we measured and TLR-mediated TNF-α responsiveness. There were no significant relationships with body size, or with indexes of general condition including BW residuals on body length, liver weight (LW) residuals on body length and PCV. This was with the exception of an isolated significant positive association between PCV and TLR9-mediated response. Nor was there any association of TLR-mediated responses with life history stage. It therefore seems unlikely that the causality for the patterns reported above was a tendency for weak or reproductively active animals to make weaker immune responses leading to higher infection levels. Whilst other authors have reported synergistic links between declining condition, infection and immunity in wild rodents [43,51], our failure to link TLR-mediated TNF-α responses and condition may be due to relatively benign environmental conditions over the sampling period, or to a lack of sensitivity of our cross-sectional survey when compared with longitudinal or interventional studies. A further possibility is that inter-host variation, independent of the measures of condition that we utilised, may have caused the observed link between TNF-α responses and parasitism. Perhaps the most likely source of such variability would be genetic polymorphisms affecting the intensity of TLR-mediated responses. Whilst mutations in individual TLR genes have sometimes been reported to affect cytokine responsiveness and resistance to infection [52-57], the strong positive covariation amongst different TLR responses in our study population would more likely relate to polymorphism in mediators affecting shared MyD88-dependent signalling pathways [58,59], or governing the expression of TNF-α [60]. If genotypic variation in wild mammal populations underpinned key phenotypic variation in the innate immune response and this, in turn, determined susceptibility to parasitism, then pinpointing the loci involved would be of fundamental interest.

## Conclusion

TLR-mediated responses represent attractive measurements for ecological studies in immunology because of their central importance in initiating innate and adaptive immunity and their modulation by signals from regulatory elements of the immune system [61]. In practice, many individual *A. sylvaticus *produced measurable TLR-mediated TNF-α responses, and there was considerable variation in responsiveness between individuals. Viewed in the context of published experimental studies on infections in *M. musculus*, the present results in a natural *A. sylvaticus *population suggest that a subset of the metazoan parasite community may be important mediators of the level of innate immune activation. A systemic down-regulation of TLR activity by parasite-induced DC-driven T_reg _expansions seems a plausible mechanistic explanation for the current observations, although immunomodulation by other routes cannot be discounted. One other possibility might be modulated TLR function (tolerance [62]) induced by greater exposure to ligands during chronic infection states. Whatever the mechanism, the existence of parasite-driven effects on innate mediators that trigger immune responses against a spectrum of different infectious agents provides strong, albeit indirect, evidence that pathogen/parasite communities may be structured by cross-species effects on innate immune function. This is highly relevant to the current interest in the importance of interspecies interactions in pathogen community dynamics [17,63]. The profound dampening of innate immune responsiveness associated with some metazoan parasite infections in *A. sylvaticus *also supports a view that modern parasite-free human and domesticated vertebrate populations may exist at levels of innate immune activation much greater than would have been typical during their recent evolutionary history. The operation of immune systems in this unnatural configuration may uncover maladaptations in control networks that were neutral in the historical context of high metazoan parasite exposure/high regulatory activity [64]. Our results are consistent with recent observations that gastrointestinal nematodes have regulatory effects on adaptive responses in laboratory models [18] and exposed human populations [9]. Importantly, we also make the novel observation that lice may be a strong source of immunoregulatory stimuli that is absent in many modern human populations.

## Methods

### Study site

Sampling was carried out in July–November 2007 at Cotgrave forest, an area of about 135ha of coniferous and mixed forestry plantation in Nottinghamshire, UK (+52.891, -1.041242). Mice were obtained by overnight trapping using variable numbers of Longworth traps assigned randomly amongst 80 permanently marked stations (1 or 0 traps/station). Stations were located at 20 m intervals along a 1600 m linear transect extending through the margins of contiguous forestry blocks. This protocol was intended to spread sampling effort evenly over a relatively large time and distance, so as not to place very high levels of mortality on the study population. Trapping rate was equivalent to 12.5 *A. sylvaticus*/km/month.

### Animals

Traps baited with peanut butter were set overnight (17.00) and collected the following morning (08.00). Captured animals were returned to the laboratory, weighed (g) and housed in identical individual cages under a lighting regimen equivalent to the natural photoperiod at the time of capture. Each cage was floored with soft wood chippings and contained sufficient soft bedding for the animal to make a nest. Each animal had free access to water and a standard selection of foods that previous trials had shown *A. sylvaticus *specimens found palatable. Approximately half of each group of animals were processed between 09.00 and 12.00 on the next day (25 to 28 hours post-capture, 'day 1'). The remainder of the group were processed at the same time on the next day (49 to 52 hours post-capture, 'day 2'). Each animal was killed by an overdose of chloroform followed by exsanguination. SVL (mm) and BW were recorded prior to exsanguination. Each animal was then dissected in a Class 2 safety cabinet and the spleen was removed to RPMI 1640. Initial parasitological observations were made on surfaces within the body cavity (see below) and the gut was transferred to 70% ethanol for dissection and microscopic examination of gastrointestinal parasites at a later time. A blood sample was collected instantly on cardiac puncture in 100 μl heparinised microcapillary tubes and spun in a microhaematocrit centrifuge (Hawksley) for 3 minutes to provide an estimate of PCV ([height of cell column/total column height] × 100). LW (wet weight, g) and reproductive status were recorded. Carcasses were wrapped in white tissue, enclosed within a plastic bag and examined later for the presence of ectoparasites (within 24 hours).

### Infection variables

We recorded all endoparasitic organisms detectable at the light microscope level in the faeces, within the lumen of the gut, within the peritoneal cavity, or visible on the surfaces of abdominal internal organs. Six metazoan parasite species occurred in the gut lumen: the cestode *Skjrabinotaenia lobata*, the digenean *Brachylaemus recurvum *and the nematodes *Heligmosomoides polygyrus*, *Syphacia stroma*, *Heterakis spumosa *and *Aoncotheca annulosa*. Individual counts were made for each of these species. For *S. stroma*, model analyses are presented for the number of established adult females worms as rapid development combined with retroinfection and/or autoinfection processes [65] may lead to great short-term fluctuation in the numbers of males and larvae present. Parallel analyses on total *S. stroma *counts suggest equivalent results (not shown). Another nematode, *Calodium hepaticum *(= *Capillaria hepatica*) occurred in the liver. Female burrows of this histozoic species are associated with prominent fibrotic reactions [66] to deposited eggs. Infection severity for *C. hepaticum *was graded according to the extent of fibrotic burrows in the liver (0: absent; 1: few fibrotic burrows in one hepatic lobe; 2: few fibrotic burrows in more than one lobe, or an extensive mass of fibrotic burrows in one lobe; 3: extensive masses of fibrotic burrows in more than one lobe). We also recorded infection with all ectoparasitic arthropods. Individual counts were made for the flea *Ctenophthalmus nobilis*, the tick *Ixodes trianguliceps *and laelapid mites. Infection with the louse *Polyplax serrata *was graded by the abundance of egg cases in the fur (0, absent; 1 <20; 2, >20). Infection with trombiculid mites was graded by the extent of the accompanying dermatitis (0, absent; 1, single local patch <5 mm in diameter). Faecal samples were examined by the McMaster flotation technique, allowing counts to be made for *Eimeria *spp. oocysts (minimum detection limit: 100 oocysts/g). Although *Eimeria *can occur in multi-species communities [67,68], and variation in oocyst morphology was observed in the present study, it was not possible to unambiguously distinguish different taxa and *Eimeria *infection is analysed here as an overall FOC.

### Immunological assay concept

TLRs are PRRs that detect pathogen-associated molecular patterns. They are of central importance in the induction of anti-pathogen immune responses, initially recognising an infection threat and mobilising inducible innate responses [11]. TLR stimulation is also crucial in shaping how antigen presenting cells activate and polarise adaptive T-helper cell responses [61]. Although TLRs may utilise more than one intracellular signalling pathway, all known human and mouse TLRs except TLR3 can signal via MyD88 resulting in the production of pro-inflammatory cytokines, including TNF-α [69]. We use an *ex vivo *stimulatory assay to detect TNF-α cytokine production by splenocytes following TLR stimulation. Our underlying assumption is that the *ex vivo *response mediated by a particular receptor against a particular agonist reflects the relative strength of the response that would occur had the same receptor-ligand interaction taken place *in vivo*. To give a profile of responsiveness in TLRs with independent specificities directed against different pathogen molecular structures [10], we measured TNF-α production mediated by TLR2, TLR4, TLR5, TLR7 and TLR9 against defined agonists (Table 1).

### Cell culture

Spleens were disaggregated through a 70 μm cell strainer into RPMI 1640. Following erythrocytic lysis (Sigma R7757), leucocytes were washed three times in RPMI 1640 and then cultured (37°C, 5% CO_2_) on 96 well plates at 2 × 10^6 ^cells/ml in RPMI 1640 supplemented with 24 mM NaHCO_3_, 10% heat-inactivated foetal calf serum, 2 mM L-glutamine, 100 u/ml penicillin, 100 μg/ml streptomycin and 60 μM monothioglycerol. Duplicate individual cultures (150 μl volume) were stimulated with one of six different TLR agonists (at the concentrations shown in Table 1) or left unstimulated as negative controls. Culture supernatants were collected after 24 hours and stored at -80°C. All receptor agonist reagents utilised were tested for functional induction of the target TLR and for endotoxin contamination by the manufacturer (InvivoGen, San Diego, CA, USA). Optimal agonist concentrations were determined by preliminary dose-response experiments on splenocyte cultures from Cotgrave *A. sylvaticus*.

### TNF-α enzyme-linked immunosorbent assay

*A. sylvaticus *is related to the laboratory models *M. musculus *and *Rattus norvegicus *at the subfamily level (Murinae), with recent molecular analyses leaving phylogenetic relationships amongst the three taxa unresolved [70] or tentatively placing *Apodemus *spp. closest to *Mus *[71]. Although the TNF-α gene and protein in *A. sylvaticus *has not been characterised, a comparison of TNF-α amino acid sequences in *R. norvegicus *(GenBank NP_036807.1) and *M. musculus *(NP_038721.1) indicated a similarity of 94%. As some possibility of cross-reactivity of antibody reagents might reasonably be expected at amino acid sequence similarities of >85%, and *A. sylvaticus *is unlikely to be much more diverged from *M. musculus *than *R. norvegicus*, we investigated the possibility that commercially available reagents for the detection of murine TNF-α might be applicable to *A. sylvaticus*. A standard anti-*M. musculus *TNF-α sandwich enzyme-linked immunosorbent assay (ELISA) (R&D, DY410) was identified in which the capture and detection antibody reagents were found to cross react with supernatants from *A. sylvaticus *splenocyte cultures subjected to a pro-inflammatory stimulus. Preliminary analyses (not shown) further confirmed that ELISA measurements in supernatants from splenocyte cultures exposed to a range of pro-inflammatory agents (HKLM, *Escherichia coli *K12 lipopolysaccharide, *Salmonella typhimurium *flagellin, FSL-1, imiquimod, ssRNA40, ODN2006; InvivoGen) increased in a dose-dependent manner. Duplicate supernatants for each assay condition in the main study (see above) were analysed on 96-well microplates (following manufacturer's instructions), with a seven-point recombinant mouse TNF-α (31 to 2000 pg/ml) standard curve run in duplicate on each plate. ELISA plate layout mirrored that of the culture plate, so that a single term for 'assay plate' could be included in statistical analyses to account for non-independence introduced into the data by the assaying procedures. Given that the ELISA antibody reagents are likely to have a differing affinity to *M. musculus *and *A. sylvaticus *TNF-α, the results we report above may be approximate estimates of *A. sylvaticus *TNF-α concentration, but should reflect relative variation between individuals. As few individuals showed detectable responses in control (unstimulated) cultures and these responses were low, data from the stimulatory conditions are analysed as the determined pg/ml readings rather than as an above-control value (analyses on data with control concentrations subtracted gave equivalent results, not shown).

### Measures of host status

In addition to PCV, we also used residuals from regressions of BW and LW on SVL as derived measures of host condition. In each of these cases a linear regression was a good fit to the data. Individual hosts were classified in the following life history stage categories: 1, juvenile male (non-scrotal with undeveloped testes); 2, non-reproductive adult male (non-scrotal with small testes and non-expanded seminal vesicles); 3, reproductive adult male (scrotal with large testes and expanded seminal vesicles); 4, juvenile female (imperforate, pelvis closed); 5, non-pregnant, non-lactating adult female (perforate and/or pelvis open); 6, pregnant female.

### Data analysis

Some missing values occurred for two study animals, so that all multivariate analyses and some analyses for individual TLR responses are based on N = 98 or 99. Where a logarithmic transformation is used, this is log_10 _(x +1). PCA on the correlation matrix was used to analyse correlational structure amongst log-transformed immunological variables. PCA of parasite data included either a log-transformed count variable or an ordinal categorical variable for each of the nine commonest species (prevalence ≥ 20%). The significance of the largest first component (PC1) extracted from PCAs was assessed by a previously described randomisation test [72]. The strength and direction of the contributions of individual variables to a principal component relate to the magnitude and sign of variable coefficients on that component. Ranks-based correlation coefficients (Spearman's, *r*_*s*_) were also used to assess pairwise associations between variables. Where there was a significant first component for a PCA, suggesting redundancy amongst the set of variables analysed, individual component (PC) scores were used to represent the main axis of shared variation in subsequent analyses. This is a standard data reduction technique, where the PC scores represent a composite variable derived as the sum of the products of individual observations and variable coefficients. PC1 score variables are designated PC1^*I *^for the immunological analysis and PC1^*P *^for the parasitological analysis. Analyses of reduced data were used to minimise the number of the main hypothesis tests (H_0 _= infection variables are unrelated to TLR-mediated responsiveness), which were then followed up by exhaustive post hoc testing of individual variables [72-74]. As part of the post hoc analysis we also investigated other components from PCAs with eigenvalues >1, but these results are not shown as they failed to reveal any significant relationships involving the immunological responses.

In order to model covariation of TLR-response variables with other TLR-response variables, or with parasite variables, in the context of confounder variables and correlations introduced by the immunological assaying structure we used residual maximum likelihood (REML) mixed models (fixed model: TLR response ~sex + SVL + season + trap group + variable of interest; random model: trap day/process day/assay plate). Sex (male/female) and season (summer/autumn) were included as factors with two levels and trap group as a factor with seven levels determined by the distribution of traps within contiguous forestry blocks. Significance of terms individually dropped from the full model was assessed by Wald tests. TLR variables with zero or few non-responders (TLR2, TLR9, TIR) were treated as continuous responses in REML LMMs. For some continuous individual TLR variables log-transformation produced significant improvements in model fit and, for consistency, all significance results, unless otherwise indicated, relate to models of log-transformed TNF-α (pg/ml). In all cases these showed satisfactory fit to the data by standard model diagnostics (see below). For predictions in LMMs, analyses were based on untransformed TNF-α concentrations and relate to averaged values for other variables included in the model. For highly skewed TLR responses with around 50% zero values (TLR4, 5 and 7), data were coded to binary form (responder/non-responder) and analysed using REML GLMMs specifying binomial errors (trial size = 1). When a skewed continuous parasite variable was used as an explanatory variable in a mixed model analysis, we routinely assessed the sensitivity of the outcome to log-transformation and ordinal categorisation of that variable. In order to model pairwise covariation amongst infection variables in the context of confounder variables, we used maximum likelihood GLMs. Depending on the distributions of the response variables, we either specified Poisson or negative binomial errors with a log link function, using an adjusted scale parameter where appropriate. Only data for common parasites (prevalence ≥ 20%) were analysed, the remaining species occurring at levels that might be considered negligible. Significance in GLMs was assessed by deleting terms [75] from a model containing all confounder variables (parasite variable 1 ~SVL + sex + season + trap group + parasite variable 2). Standard model diagnostics (residual distributions, residual versus fit plots, normal and half-normal plots) were used to assess the fit of GLMs, LMMs and GLMMs to the data. PCA was implemented in Minitab^® ^15.1.0.0 and linear models in GenStat^® ^version 10.1.0.71.

### Stress effects

Although elevated stress cannot be eliminated in studies where wild mammals are subject to handling and periods of captivity, there was no significant difference for any of the TLR-mediated response variables, including TIR, PC1^*I *^and each of the individual TLR responses, between animals analysed 1 or 2 days post-capture (LMMs or GLMMs of the form: TLR variable ~SVL + sex + season + trap group + process time, random model = capture date/assay plate; *P *> 0.05). This suggests that any stress-related immunological variation had stabilised by the time the first animals were processed.

## Abbreviations

BW: body weight; DC: dendritic cell; ELISA: enzyme-linked immunosorbent assay; FOC: faecal oocyst count; GLM: generalised linear model; GLMM: generalised linear mixed model; HKLM: heat-killed *Listeria monocytogenes*; LMM: general linear mixed model; LW: liver weight; PCA: principal components analysis; PCV: packed blood cell volume; PRR: pattern-recognition receptor; REML: residual maximum likelihood; SVL: snout-vent length; TLR: toll-like receptor; TNF-α: tumour necrosis factor alpha.

## Authors' contributions

JMB and PDH provided background information about the study system. JAJ, IMF, LB and JEB designed the study and developed the immunological assays. Fieldwork was carried out by LB and JAJ. Parasitology was carried out by JAJ. PDH assisted in the identification of ectoparasites. Immunology was carried out by JAJ, CR and AL. JAJ analysed the data. JAJ, IMF and JEB wrote the paper, with critical input from PDH and JMB. IMF carried out confirmatory work.
